# Megahertz pulse trains enable multi-hit serial femtosecond crystallography experiments at X-ray free electron lasers

**DOI:** 10.1038/s41467-022-32434-6

**Published:** 2022-08-11

**Authors:** Susannah Holmes, Henry J. Kirkwood, Richard Bean, Klaus Giewekemeyer, Andrew V. Martin, Marjan Hadian-Jazi, Max O. Wiedorn, Dominik Oberthür, Hugh Marman, Luigi Adriano, Nasser Al-Qudami, Saša Bajt, Imrich Barák, Sadia Bari, Johan Bielecki, Sandor Brockhauser, Mathew A. Coleman, Francisco Cruz-Mazo, Cyril Danilevski, Katerina Dörner, Alfonso M. Gañán-Calvo, Rita Graceffa, Hans Fanghor, Michael Heymann, Matthias Frank, Alexander Kaukher, Yoonhee Kim, Bostjan Kobe, Juraj Knoška, Torsten Laurus, Romain Letrun, Luis Maia, Marc Messerschmidt, Markus Metz, Thomas Michelat, Grant Mills, Serguei Molodtsov, Diana C. F. Monteiro, Andrew J. Morgan, Astrid Münnich, Gisel E. Peña Murillo, Gianpietro Previtali, Adam Round, Tokushi Sato, Robin Schubert, Joachim Schulz, Megan Shelby, Carolin Seuring, Jonas A. Sellberg, Marcin Sikorski, Alessandro Silenzi, Stephan Stern, Jola Sztuk-Dambietz, Janusz Szuba, Martin Trebbin, Patrick Vagovic, Thomas Ve, Britta Weinhausen, Krzysztof Wrona, Paul Lourdu Xavier, Chen Xu, Oleksandr Yefanov, Keith A. Nugent, Henry N. Chapman, Adrian P. Mancuso, Anton Barty, Brian Abbey, Connie Darmanin

**Affiliations:** 1 Department of Mathematical and Physical Sciences, School of Engineering, Computing and Mathematical Sciences, La Trobe University, Melbourne, VIC 3086 Australia; 2 La Trobe Institute for Molecular Science, La Trobe University, Melbourne, VIC 3086 Australia; 3 European XFEL, Holzkoppel 4, 22869 Schenefeld, Germany; 4 School of Science, RMIT University, Melbourne, VIC 3000 Australia; 5 Australian Nuclear Science and Technology Organisation (ANSTO), Sydney, NSW 2234 Australia; 6 Center for Free-Electron Laser Science CFEL, Deutsches Elektronen-Synchrotron DESY, Notkestr 85, 22607 Hamburg, Germany; 7 Deutsches Elektronen-Synchrotron DESY, Notkestr 85, 22607 Hamburg, Germany; 8 The Hamburg Centre for Ultrafast Imaging, Luruper Chaussee 149, Hamburg, 22761 Germany; 9 Institute of Molecular Biology, SAS, Dubravska cesta 21, 845 51 Bratislava, Slovakia; 10 Lawrence Livermore National Laboratory, 7000 East Avenue, Livermore, CA 94550 USA; 11 Dept. de Ingeniería Aeroespacial y Mecánica de Fluidos, ETSI, Universidad de Sevilla, 41092 Sevilla, Spain; 12 Department of Mechanical and Aerospace Engineering, Princeton University, Princeton, NJ 08544 USA; 13 Max-Planck Institute for the Structure and Dynamics of Matter, Luruper Chaussee 175, 22761 Hamburg, Germany; 14 University of Southampton, Southampton, SO17 1BJ UK; 15 Institute of Biomaterials and Biomolecular Systems, University of Stuttgart, Am Pfaffenwaldring 57, 70569 Stuttgart, Germany; 16 School of Chemistry and Molecular Biosciences, Institute for Molecular Bioscience and Australian Infectious Diseases Research Centre, University of Queensland, Brisbane, QLD 4072 Australia; 17 Department of Physics, Universität Hamburg, Luruper Chaussee 149, 22761 Hamburg, Germany; 18 School of Molecular Science, Arizona State University, Tempe, AZ 85281 USA; 19 Institute of Experimental Physics, TU Bergakademie Freiberg, Leipziger, Str. 23, 09599 Freiberg, Germany; 20 ITMO University, Kronverksky pr. 49, St. Petersburg, 197101 Russia; 21 Hauptman-Woodward Medical Research Institute, 700 Ellicott St., Buffalo, NY 14203 USA; 22 Department of Physics, University of Melbourne, Parkville, VIC 3010 Australia; 23 Biomedical and X-ray Physics, Department of Applied Physics, AlbaNova University Center, KTH Royal Institute of Technology, SE-106 91 Stockholm, Sweden; 24 Department of Chemistry, State University of New York at Buffalo, 760 Natural Sciences Complex, Buffalo, NY 14260 USA; 25 Institute for Glycomics, Griffith University, Southport, QLD 4222 Australia; 26 Department of Quantum Science and Technology, Research School of Physics, Australian National University, Canberra, ACT 2601 Australia; 27 Present Address: La Trobe Institute for Molecular Science, La Trobe University, Melbourne, VIC 3086 Australia

**Keywords:** Free-electron lasers, X-ray crystallography

## Abstract

The European X-ray Free Electron Laser (XFEL) and Linac Coherent Light Source (LCLS) II are extremely intense sources of X-rays capable of generating Serial Femtosecond Crystallography (SFX) data at megahertz (MHz) repetition rates. Previous work has shown that it is possible to use consecutive X-ray pulses to collect diffraction patterns from individual crystals. Here, we exploit the MHz pulse structure of the European XFEL to obtain two complete datasets from the same lysozyme crystal, first hit and the second hit, before it exits the beam. The two datasets, separated by <1 µs, yield up to 2.1 Å resolution structures. Comparisons between the two structures reveal no indications of radiation damage or significant changes within the active site, consistent with the calculated dose estimates. This demonstrates MHz SFX can be used as a tool for tracking sub-microsecond structural changes in individual single crystals, a technique we refer to as multi-hit SFX.

## Abstract (web summary)

Free-electron lasers are capable of high repetition rates and it is assumed that protein crystals often do not survive the first X-ray pulse. Here the authors address these issues with a demonstration of multi-hit serial crystallography in which multiple FEL pulses interact with the sample without destroying it.

## Introduction

The advent of Serial Femtosecond Crystallography (SFX) has created opportunities via which macromolecular structures can be probed and their dynamics investigated. SFX is ideally suited to studying the molecular dynamics of molecules undergoing irreversible processes which cannot be measured using conventional synchrotron or lab-based X-ray sources^1–7^. One practical hurdle to implementing SFX is obtaining a large enough data set for high-resolution 3D structure determination which typically comes at the cost of high sample consumption. The first generation of X-ray Free Electron Laser (XFEL) facilities typically had pulse repetition rates of the order of 120 Hz or less meaning that obtaining a large enough data set for structure retrieval is an inefficient process both in terms of the amount of sample required but also in terms of the amount of XFEL beamtime needed^8^. With the development of high-repetition rate sources like the European XFEL^9^ both data collection times and sample consumption are significantly reduced^1,10^. Another avenue of research open to MegaHertz (MHz) XFEL facilities is the potential to use the unique pulse structure of these sources to perform time-resolved experiments^9,11,12^. Typical Time-Resolved SFX (tr-SFX) experiments are performed either by optical pump/X-ray probe reactions initiated by an optical laser, or mix-and-inject experiments initiated via solvent diffusion in the crystal^13^. A split-and-delay method also enables timing regimes of 20 fs – 100 fs to be accessed in order to probe molecular dynamics^14^. The ultra-short pulse duration of the XFEL supports the study of in-situ molecular dynamics on sub-picosecond timescales by measuring a large number of crystals at various pump-probe delay times or mixing times^15–19^. In addition, to probing ultra-fast molecular dynamics, these measurements normally result in diffraction before destruction, where each crystal is measured once before being destroyed.

The time delay between two consecutive pulses at MHz XFEL sources is normally within the micro to nanosecond range. This creates an opportunity to explore a range of molecular dynamics occurring on sub-microsecond timescales by using consecutive pulses in the train (which are typically on the order of 10’s fs) to capture multiple diffraction patterns from the same crystal as it traverses the X-ray beam. Previously, multiple hits of the same single crystal using XFELs have only been detected using high-viscosity jet streams and a static chip system. This is because, unlike liquid jets, high-viscosity jets can flow at speeds slow enough that even at lower (e.g., non-MHz) repetition rate XFEL facilities and synchrotrons can observe multiple hits on the same crsytal^20–23^.

Analysing multi-hit serial crystallography data collected using liquid jets, makes it possible to study time-resolved molecular dynamics within individual crystals enabling us to differentiate between two crystal states within the one crystal. For example, when using an optical pump/X-ray probe system, the dark state and optically activated ‘pumped’ state can be measured on the same crystal consecutively enabling the exact correlation of molecular information at a precise time interval. This allows us to potentially detect a protein movement within the same crystal on a submicron time scale. In chemical mix-and-inject experiments in which the distance along the jet of the X-ray beam from the mixing region determines the initial time point, the timing between consecutive pulses determines the timescale of the molecular dynamics that can be probed within a single crystal. The advantage of multi-hit serial femtosecond crystallography (multi-hit SFX) over conventional tr-SFX is that at least two diffraction patterns can be collected from a single crystal at different time points, within the microsecond to sub-microsecond time regime, making it easier to track/correlate any changes in the molecular structure. These time scales allow us to probe the molecular dynamics of proteins, more specifically ligand binding, domain folding, transition states (i.e., switching between the active and inactive form)^24–28^, helical motion^29^ and side-chain rotations^30^. The combination of femtosecond intra-pulse and sub-microsecond inter-pulse timing resolution is something that is specific to MHz XFEL sources. The feasibility of performing experiments that exploit both characteristics is the topic of the present paper.

An example problem that could benefit from this type of multi-hit SFX is understanding the mechanism of the Bacillus subtilis response regulator, SpoOF, which is involved in sporulation. SpoOF can induce a shift in protein conformation on picosecond to millisecond timescales^27,31^. Previous NMR studies have demonstrated the secondary structure dynamics of the protein involves a complex series of movements including rotation of bonds in methyl groups (thought to occur within nanoseconds) and side chain flipping of buried residues (which occurs on the scale of seconds). Critical protein-protein contacts between SpoOF and its binding partners meanwhile take place on millisecond time-scales^27^. Therefore, multi-hit SFX studies on the same crystal could be used to probe the intermediate states between the protein-protein interactions in SpoOF and the flipping of residues to accommodate it. Other example applications that will benefit from multi-hit SFX include studying protein-protein interactions e.g., determining the initial binding interactions involved in the adaptor protein oligomerisation of myeloid differentiation primary response gene88, which would aid in understanding its role in immunity^7^. In addition, studying the binding of co-factors such as ATP or NADP, which induce extensive conformational changes within proteins and result in the transition between active to inactive forms of the enzyme, is known to occur on millisecond timescales and will also be a target for multi-hit SFX. It could also be used to capture the intermediate helical/sheet conformational changes in the secondary structures of proteins prior to side chain flipping events. Thus, multi-hit SFX will enable the capture of intermediate states in-situ based on the inter-pulse timing, which can be varied within a single pulse train^32^.

Currently, the most common method for delivering samples to the XFEL beam is via a liquid jet, formed using a flow focusing nozzle or Gas-focused Dynamic Virtual Nozzle (GDVN)^33–35^, in which the sample is continuously replenished. Typically, no single crystal contributes more than one diffraction pattern to the dataset, either because it is destroyed during interaction with the XFEL or because it has moved out of the interaction region prior to the next X-ray pulse arriving^36–38^. However, for crystals which are micron-sized or larger, it is possible to obtain high-resolution, time-resolved structural data at an XFEL using an X-ray beam that is much larger than the dimensions of the crystal^38^. Crystals of this size are typically used for tr-SFX experiments where the primary motivation behind using the XFEL is the short pulse duration which enables snapshots to be taken of dynamically evolving molecular structures^13^. The combination of femtosecond intra-pulse and sub-microsecond inter-pulse timing which is now accessible at the European XFEL thus creates an opportunity to explore molecular dynamics on sub-microsecond timescales by varying the inter-pulse spacing.

However, it is important to note, that for the same crystal to be hit twice during a multi-hit SFX experiment, the dose received by the crystal from the first hit should be below the Room Temperature (RT) damage limit. This can be estimated prior to the experiment (e.g., using programs such as RADDOSE^39^) and confirmed during the experiment by comparing, on-the-fly, the diffraction data collected during the first and second hit. We also note here, that the multi-hit diffraction scenario is clearly distinct from multi-crystal diffraction, in which two or more crystals arrive at the X-ray beam at the same time, and which can be identified in the diffraction pattern and by using multi-crystal indexing options^40–42^. Whilst this mode of data collection likely precludes the use of nanocrystals due to their weak diffraction, it does allow for multi-hit, femtosecond diffraction experiments to be performed on microcrystals which are commonly used for tr-SFX^16,43^.

In this work we present, an experimental investigation comparing XFEL derived protein structures, from the same crystal, measured in consecutive pulses using continuous liquid jets. Using the European XFEL data collected at different liquid jet flow rates, (40–102 m/s), we obtain separate high-resolution datasets from the first and second hits on the same single crystal, hit twice, in a MHz SFX experiment. Multi-hit SFX, can be thought of as a bridge between SFX and conventional crystallography; in SFX typically thousands of crystals are hit once and destroyed by the XFEL beam, whereas in conventional crystallography one crystal is continuously rotated, and multiple diffraction patterns are collected prior to significant radiation damage occurring. Here we show how often multi-hits (in this case double hits) are observed in the MHz SFX experiments; why multi-hits occur and what is the influence of the experimental geometry; how the data quality and resolution of the first and second hits compare and provide experimental conditions optimised for multi-hit SFX to help tailor future MHz XFEL experiments. Both an analysis of the measured diffraction intensities from the two datasets and the resulting structure do not reveal any signs of radiation damage. This is consistent with RADDOSE-3D (XFEL version)^39^ calculations showing that, under the experimental conditions used here, the crystal received less than half the dose (0.106 MGy). We have also established that even larger beam sizes are readily achievable at the European XFEL whilst providing, for micron-sized crystals, enough photon flux to generate high-resolution diffraction data. Hence, it is possible, to further optimise the experimental setup to significantly increase the number of single crystals which are hit multiple times. In addition, depending on the breakup of the jet, it may even be possible to collect more than two diffraction patterns per single crystal prior to the sample either being damaged or exiting the beam (as indicated in our model) if the experimental setup (crystal size, sample flow rate, and beam size) is optimised.

## Results

### Identifying consecutive hits for a single lysozyme crystal

Assessment of the frequency of multi-hit single crystals used X-ray data corresponding to three different jet speeds (where most of the data was collected); the statistics are summarised in Table 1. The number of images collected is compared to the number of images with hits detected by Cheetah^44^ and indexed by CrystFEL^44,45^, as well as the percentage of those patterns indexed where crystals were hit twice as they passed through the beam. A crystal was determined a multi-hit, if it satisfied the following criteria: (i) the two hits were from consecutive pulses and (ii) the patterns showed very similar crystallographic orientations where the angle between each pair of basis vectors was <5° and the lengths of the basis vectors were in agreement to within 10%, determined using CrystFEL’s Whirligig program^23,44,45^. The data shows that, as expected, when the jet speed increases the percentage of crystals which were hit twice by the beam decreases. For the slowest (42 m/s), intermediate (78 m/s), and fastest (102 m/s) jet speeds the corresponding multi-hit percentages were 6.4%, 0.9% and 0.3%, respectively.

**Table 1:** Summary of jet speeds, experimental conditions, and statistics for lysozyme crystals^1^

Target jet speed	50 m/s	75 m/s	110 m/s*
Liquid flow (µl/min)	15	13	13
Gas flow (mg/min)	23	50	85
Experimental jet speed (m/s)	42 ± 2.1	78 ± 3.9	–
Theoretical jet speed (m/s)	–	–	102 ± 5.1
Total no. frames	440,000	60,000	240,000
No. of hits	10,726 (2.4%)	1,638 (2.7%)	3,733 (1.6%)
No. of indexed frames	9,970 (93%)	1,509 (92.1%)	3,474 (93.1%)
No. double hits	1190	28	20
No. single hits	8780	1481	3454

This table shows the flow rates of the gas and liquid used as well the experimentally and theoretically determined jet speeds for the current analysis. The data statistics were calculated utilising the CrystFEL software suite^44,45^.

* A 100 m/s jet speed was determined experimentally for a 13 µl/min liquid and 80 mg/min gas flow rate giving a speed of 105 m/s, which is similar to the theoretically calculated 102 m/s jet speed.

### Crystal rotation and Bragg peak analysis

**Fig. 1:**
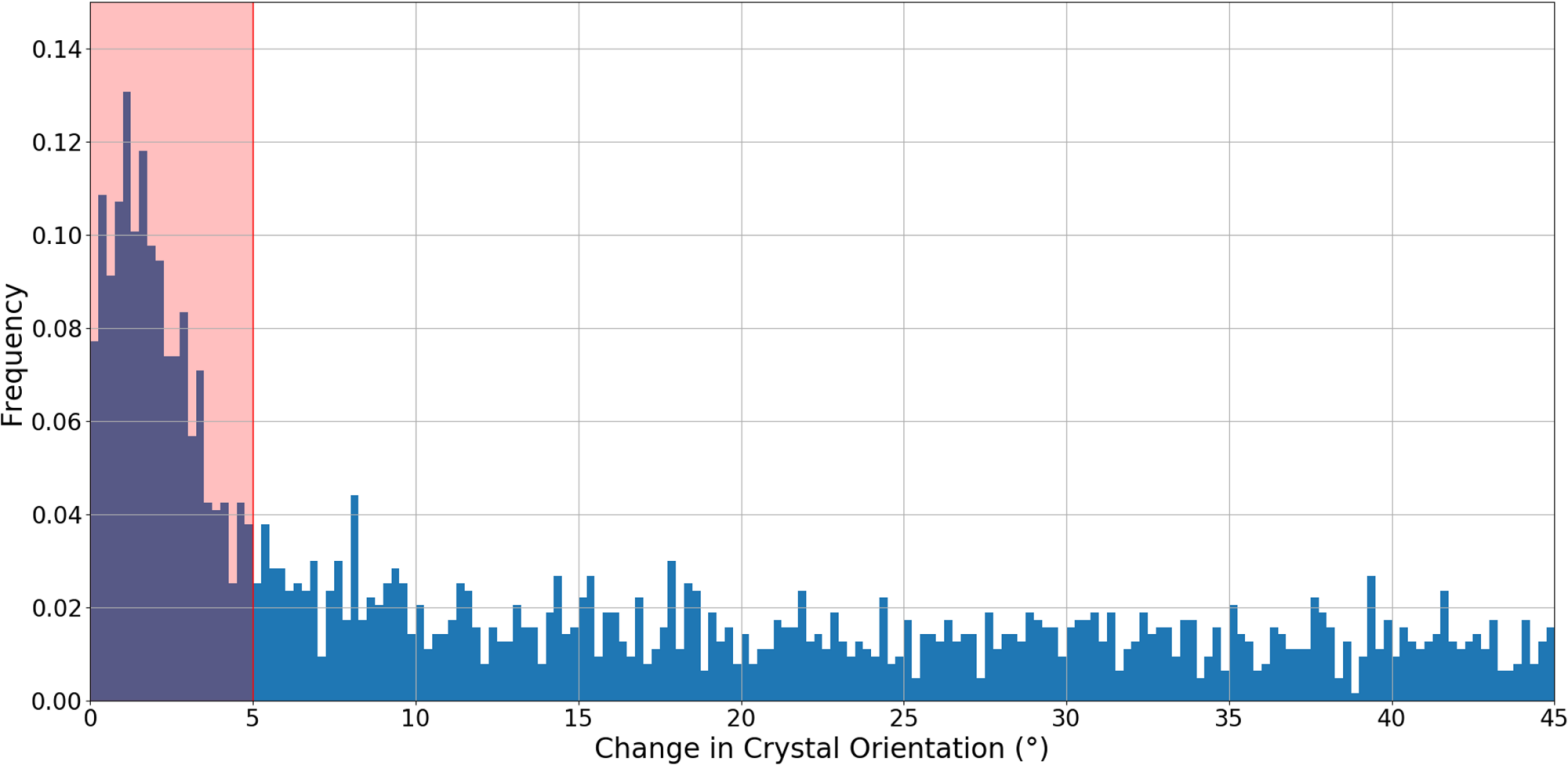
Frequency of diffraction patterns as a function of the relative change in crystal orientation. The change in crystal orientation was characterized by the reciprocal space vector $\mathop{a}\limits^{ \rightharpoonup }$, between consecutive diffraction measurements (separated in time by 886 ns) within the X-ray pulse train. The liquid jet speed was 42 m/s. An increase in frequency above 0.04 for consecutive images with a change in orientation of less than 5 degrees, indicated by the region shaded in red, can be observed and were classified as double hit crystals.

To confirm that our method for determining which crystals were hit twice is reliable for the three jets speeds in Table 1, the change in crystal rotation for all the diffraction data collected from consecutive hits in the pulse trains was calculated. Rotation of the crystal as it tumbles in the liquid jet between consecutive X-ray pulses results in a small change in the position of the Bragg peak as illustrated in Fig. 1. A significant increase in the number of crystals with <5° change in orientation between consecutive hits was observed for the 42 m/s jet speed (Fig. 1 and Supplementary Figs. 1, 2). This indicates that many of the crystals that apparently have only a very small change in their orientation are in fact the same crystal hit twice by the XFEL beam. By contrast, the number of crystals with a change in orientation of >5° between consecutive hits does not vary significantly, consistent with crystals arriving at the X-ray interaction region with a random orientation. These results verify the predictions of the Whirligig program^45^; the same approach was also used to determine the number of multi-hit crystals that occurred for the two faster jet speeds. Due to the lower hit rates for the two faster jets speeds (78 and 102 m/s) the number of double hit diffraction patterns is much less than for the 42 m/s jet speed (see Table 1).

### Understanding multi-hit SFX as a function of jet speed

**Fig. 2:**
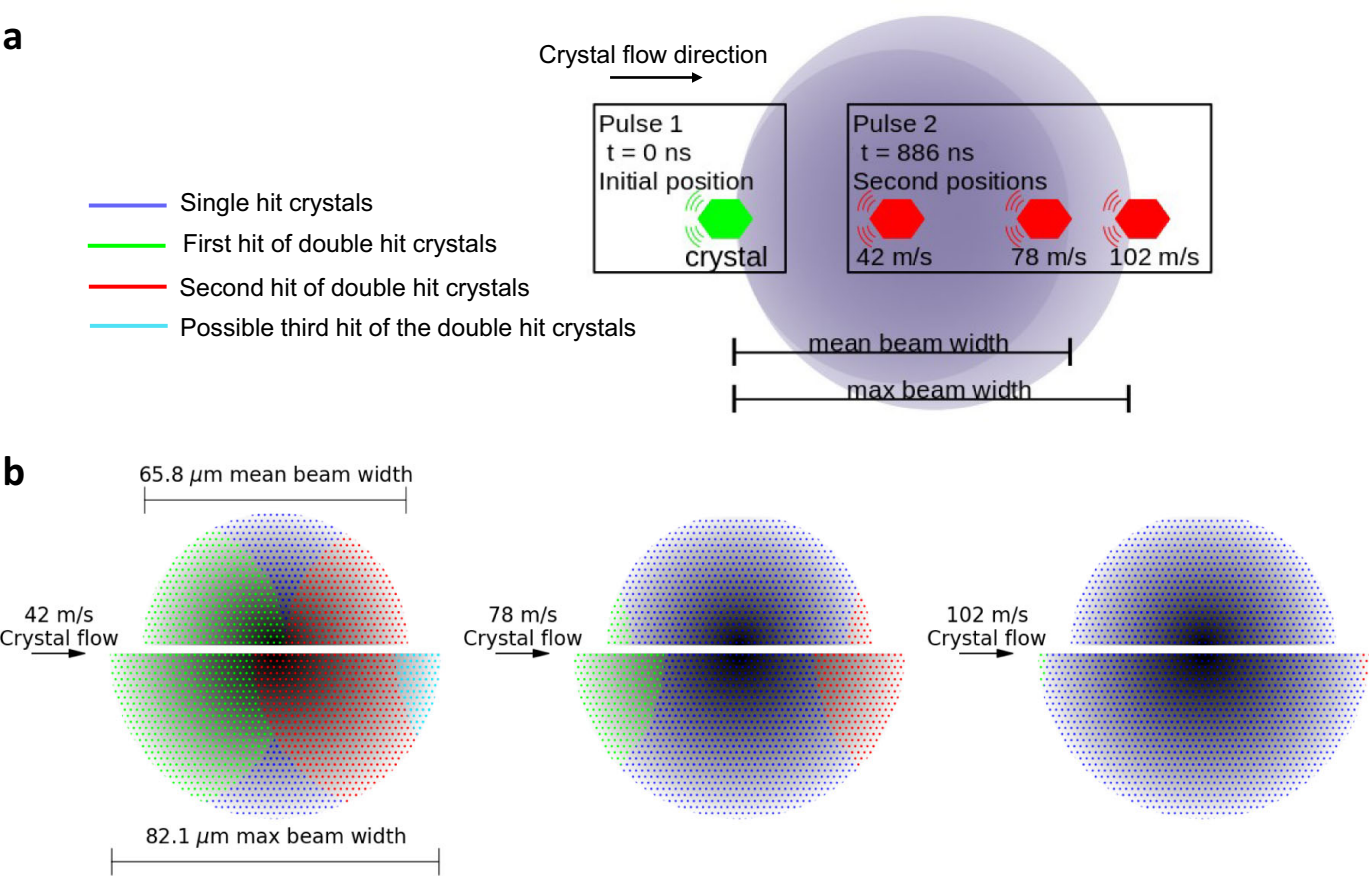
Model for how multi-hits occur for a single crystal. **a** Schematic diagram (not to scale) illustrating the minimum distances travelled by an 8 µm crystal for the three different jet speeds overlaid with the average beam Full Width (FW, dark purple shaded region) and the maximum beam FW (light purple shaded region). The green crystal depicts the initial position, and the red crystal illustrates how far the crystal travels after the first hit for 42 m/s, 78 m/s, and 102 m/s jet speeds. **b** Schematic representation of the crystal path through the X-ray beam for each of the three jet speeds for the mean beam FW (upper half) and maximum beam FW (lower half) as indicated on the first image. The mean FW is consistent for all jet speeds. The beam profile (shaded grey) is overlaid with the regions that the crystal travels through for the single hits (blue) to occur as well as the first (green) and second (red) hits of the double hit crystal. For the 42 m/s jet speed it also shows a possibility of the crystals being hit a third time (aqua) if the crystal and beam conditions were optimal. Note, for 42 m/s and 78 m/s, regions where no hits occur are possible.

Irrespective of crystal concentration, multiple hits on a single crystal are primarily determined by the jet speed, beam size, and pulse repetition rate. To illustrate how multiple hits occur, a graphical representation of the crystal path through the beam based on the experimental data is presented in Fig. 2. For the slowest jet speed (42 m/s) the crystal can be initially hit within the tail (lowest intensity) region of the X-ray beam followed by a second hit within the Full Width at Half Maximum (FWHM) of the beam. The exposure of the same crystal to a second XFEL pulse within the most intense part of the X-ray beam means that the crystal may absorb sufficient dose that it is either destroyed or no longer diffracts to high enough resolution to generate a third hit, however, this is dependent on the beam properties and the dose the crystals receive during the first two hits. Theoretically, if the dose received by the crystal, when placed centrally within the FWHM, is below the dose threshold and the speed the crystal is travelling is slow enough and the beam is large enough, there is a possibility that a third hit could occur. However, this needs to be further investigated as it would likely only be possibly for jet speeds slower than 42 m/s. As the jet speed increases to 78 m/s, multiple crystal hits are only possible within the tail regions of the X-ray beam (Fig. 2). At the fastest jet speed (102 m/s), multiple crystal hits can only occur in the extreme tail regions of the beam where the incident intensity is low compared to the central region (Fig. 2). Therefore, for the two faster jets it limits the hit number per crystal to two.

### Crystal transit through the X-ray beam

**Fig. 3:**
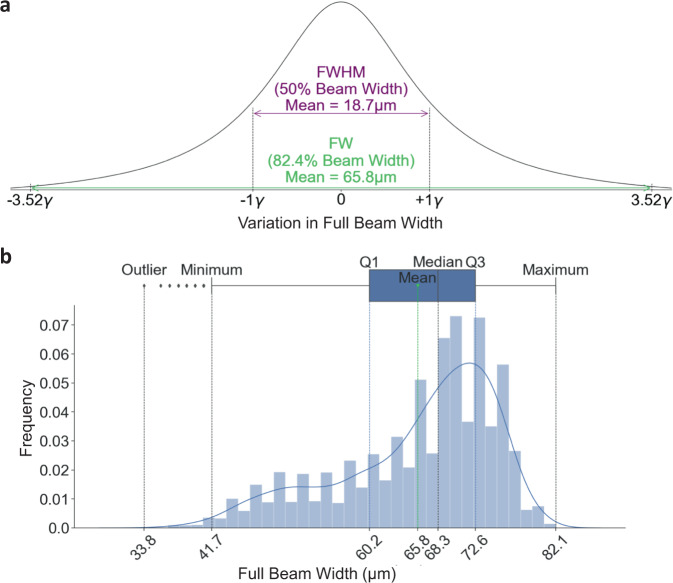
Characteristic beam profile. **a** A histogram showing the X-ray beam profile. The beam profile was modelled using a Lorentzian distribution with a Full Width Half Maximum (FWHM) = 2γ (50% of beam) and Full Width (FW) = 7.04γ (82.4% of beam). **b** The Lorentzian distribution used to determine the FWHM and FW from 6773 YAG images. The furthermost outlier, minimum, Q1 (25^th^ percentile), mean, median, Q3 (75th percentile), and maximum have been indicated. To obtain the FWHM and FW for each YAG image, a 7.5% noise threshold was applied to the image combined with a 3 × 3 median filter to account for the noise.

Assuming that at least 1 µm of the crystal needs to interact with the X-ray beam to generate a diffraction pattern and a maximum crystal size of 8 µm, the minimum distance travelled by a single crystal hit twice by the XFEL beam in this experiment was 35.4 µm, 56.7 µm, and 79.9 µm, for jet speeds of 42 m/s, 78 m/s, and 102 m/s respectively. Figure 2 presents a schematic showing how far the crystal travels between consecutive pulses for each jet speed. While Fig. 3 provides the characteristic beam profile, based on YAG images, showing the Full Width (FW) and FWHM of the X-ray beam during the experiment. The mean, minimum, and maximum, FWs were calculated to be 65.8 µm, 41.7 µm and 82.1 µm, respectively. While the FWHMs were calculated to be 18.7 µm, 11.9 µm and, 23.3 µm, respectively. This analysis confirms that for each of the three jet speeds, the X-ray beam diameter was sufficiently large that crystals are hit at least twice by the X-ray beam, which is consistent with the experimental observations.

### Data quality check

**Fig. 4:**
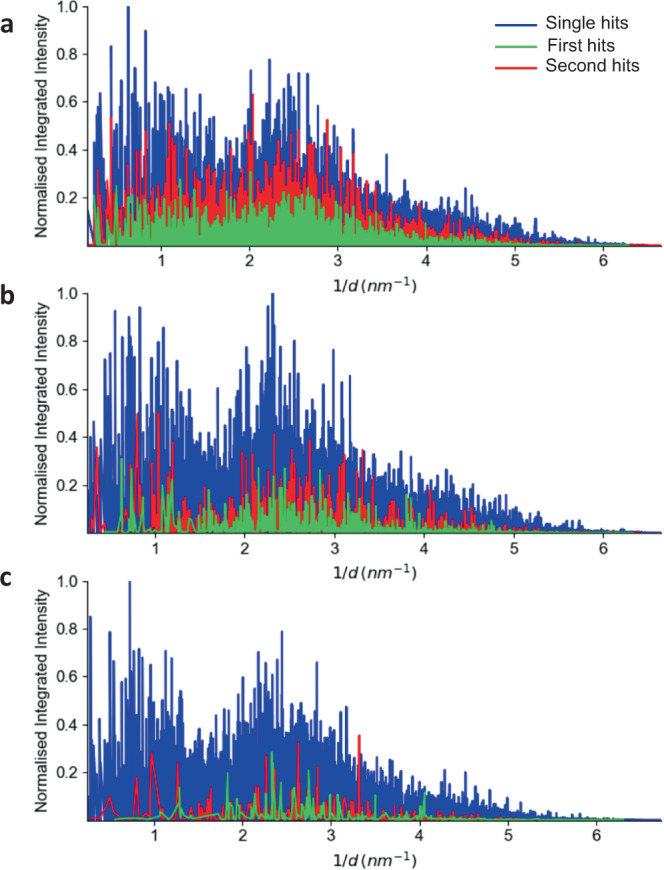
Normalized integrated intensity plots. Integrated intensities were extracted from the data, normalized and plotted against 1/*d* (where *d* is the lattice spacing) for the **a** 42 m/s jet data; **b** 78 m/s jet data; and **c** 102 m/s jet data. Blue represents data for single hit crystals only; green represents the first hit of the double hit crystal; red represents the second hit of the double hit crystal. A threshold of I/sig(I) > 2 was applied to the analysis.

In addition to calculations of the absorbed dose, the data quality was carefully analysed to confirm that there was no degradation of the diffraction patterns collected from the second hit with respect to the first. Initially, the Bragg peak intensities and resolution of the diffraction data collected from the first and second hits were compared. This data was used to generate three independent powder plots (first hits, second hits, and single hits) for the three jet speeds (42, 78 and 102 m/s). For the slowest jet speed (42 m/s), we observed that the integrated intensity of the first hit was less than the second hit (note that the intensity of the second hit was, as expected, similar to that of the single hit crystals, see Fig. 4a). As the jet speed increases, the diffraction intensity profiles between the first and second hits become similar (see Fig. 4b, c) but substantially lower when compared to the single hit crystals. This is consistent with our interpretation of how double hits occur (Fig. 1) since our model predicts that for the faster jet speeds both first and second hits occur only within the tail region of the X-ray beam.

**Fig. 5:**
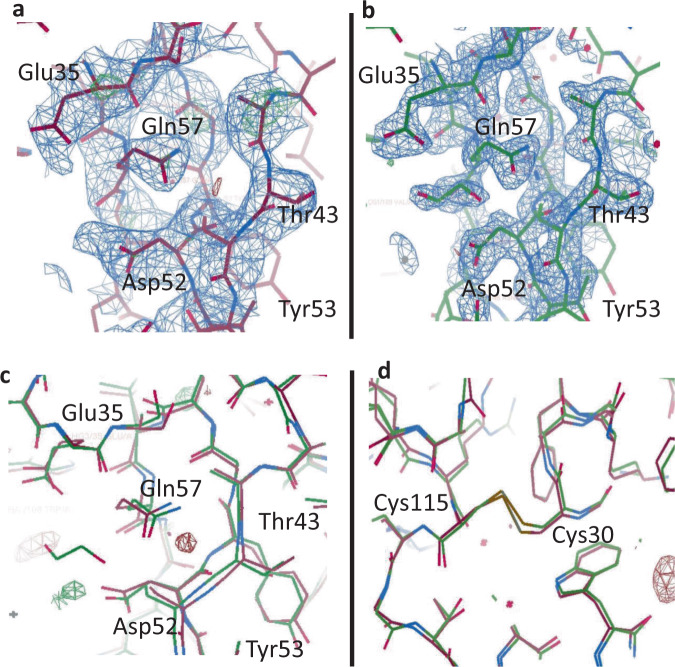
Lysozyme structural maps showing the active site pocket. The electron density map with the omit map displayed for the active site region of lysozyme in **a** the first hit structure (7TUM) and **b** the second hit structure (6WEC). **c** Shows the first (red) and second (green) hit structures superimposed and overlaid with the difference electron density (DED) map for the active site and **d** shows the DED maps for a representative di-sulphide bond Cys115-Cys30. No differences density is detected between the first and second hit structures. The 2Fo-Fc map at 1σ is shown in blue and difference maps at 3σ are shown in green (positive) and red (negative) density.

The final check was to perform a complete structure retrieval based on the independent data sets collected from the first and second hits and analyse the resulting electron density maps. A comparison of the Difference Electron Density (DED) maps from the first (PDB code 7TUM) and second (PDB code 6WEC) hit structure is presented in Fig. 5. The site specifically in the local vicinity of the active pocket of lysozyme, known to be sensitive to the effects of radiation damage, is shown in Fig. 5a–c and Supplementary Fig. 6. Superposition of the two structures, generated independently for the first and second hit data sets, does not reveal any significant differences in the backbone (rmsd of 0.3893 Å). The time-resolved DED maps (i.e., 886 ns apart) calculated the difference between the first hit and second hit structure factors and indicated that no significant changes could be attributed to the time-delay between the first and second hit on the same crystal (Fig. 5a and Supplementary Fig. 6). Crucially, examination of the di-sulphide bonds (Fig. 5d and Supplementary Fig. 6), which are very prone to radiation damage, did not result in presence of DED maps surrounding the atoms. Hence, based on these maps we conclude that there is no evidence of radiation damage occurring between first and second hits, meaning that any significant changes that did occur in a multi-hit SFX experiment performed under these conditions could be attributed to actual molecular dynamic events.

A statistical comparison between the three data sets is shown in Tables 2 and 3. The Wilson plots and CC* for all data sets are included in the Supplementary Information (Supplementary Fig. 4). Interestingly, the second hit data set quality is slightly better compared to the first hit data set which is reflected in its overall structural resolution, number of unique reflections and the CC values. The first hit data set is of poorer quality, which is likely due to the non-uniform beam shape. The more the crystal is exposed within the beam the more intense the peaks are, and this can be considered proportional to the resolution (where the most intense peaks are at lower resolution and the higher resolution are often less intense). Therefore, if the beam has a longer weaker tail at one end, diffraction from the crystal is much weaker, with peaks below the minimum Signal-to-Noise-Ratio (SNR) threshold (see Methods) remaining undetected. This, in turn, determines the number of unique reflections identified in the highest resolution shell. This can be seen in the YAG images (Supplementary Fig. 3b) where the tail extends further out on the first hit side (i.e., in the direction of the nozzle) having less intensity compared to the tail region on the opposing side where the second hit region is located. As the stream files were obtained after hit-finding and indexing, the SNR threshold was not optimised for detection of the weaker, first hits, and this may explain why we observe a reduced number of unique reflections in the first hit data set (2072) compared to the second hit data set (7263). The CC* is a commonly used metric to assess data quality for structure determination^46^. A comparison between the CC* for all three data sets reveal that the data quality varies between the different jet speeds which is reflected in the resolution. The first hit data had the lowest CC* value with a reduction in data quality observed above 3.0 Å resolution (Supplementary Fig. 4d), therefore a 3.1 Å resolution cut off was used for the structure refinement. With this cut off, the CC* values for the first and second hits were comparable; 0.873 and 0.764, respectively (Table 2). CrystFEL^45^ was also used to compare the structure factor of the first and second hit data sets to compare their quality. For the quality comparison, the first and second hit data sets were split in half; the half dataset for the second hits was then compared to the other half dataset from the first hits and vice versa and this resulted in stable CC* values up to a resolution of 2.5 Å (Supplementary Fig. 4d, green line), confirming there is good agreement between the two datasets. Further to this, the single hit data set (i.e. data from crystals only hit once) was also split and compared to the first and second hit data showing a similar degree of consistency in terms of data quality (Supplementary Fig. 4d, purple line). Hence, aside from the increase in the number of reflections in the second hit data, resulting in higher resolution data due to the higher incident intensity, the data quality between first, second, and single hits was consistent.

**Table 2:** SFX data collection and processing statistics for lysozyme

Data Set	Single hit	First hit	Second hit
Diffraction source	European XFEL	European XFEL	European XFEL
Photon Energy (mean value, eV)	9232	9232	9232
Pulse energy at sample (assuming 50% beamline transmission, µJ)	290	290	290
Wavelength (Å)	1.3	1.3	1.3
Temperature (K)	293	293	293
Detector	1-megapixel AGIPD	1-megapixel AGIPD	1-megapixel AGIPD
Pulse length (fs)	50	50	50
Space group	*P*4_3_2_1_2	*P*4_3_2_1_2	*P*4_3_2_1_2
*a*, *b*, *c* (Å)	79.30, 79.30, 37.73	79.30, 79.30, 37.73	79.30, 79.30, 37.73
α, β, γ (°)	90, 90, 90	90, 90, 90	90, 90, 90
Resolution range (Å)	21.66-2.10 (2.15-2.10)	35.49-3.20 (3.28-3.20)	21.66-2.10 (2.15-2.10)
Indexed	10,106	962	962
No. of unique reflections	7,418 (535)	2072 (403)	7,263 (494)
Completeness (%)	99.84 (100)	93.6 (90.79)	97.75 (92.34)
Redundancy	47.24 (28.34)	4.55 (3.8)	6.66 (3.98)
〈 *I*/σ(*I*)〉	5.1 (4.2)	4.36 (8.3)	2.9 (3.1)
CC_1/2_	0.906 (0.796)	0.412 (0.638)	0.615 (0.413)
CC*	0.975 (0.942)	0.764 (0.882)	0.873 (0.764)
Overall *B* factor from Wilson plot (Å^2^)	19.58	32.8	20.4

Statistics for the single crystal as well as the multi-hit (first and second) data sets are presented. Values for the outer shell are given in parentheses.

**Table 3:** SFX and refinement statistics for lysozyme

Data Set	Single Hit	First Hit	Second Hit
Resolution range (Å)	21.66–2.10 (2.15–2.10)	35.489–3.20 (3.28–3.20)	21.66–2.10 (2.15–2.10)
Completeness (%)	99.84 (100)	93.6 (90.79)	97.75 (92.34)
No. of reflections, working set	6681 (737)	1850 (129)	6547 (716)
No. of reflections, test set	479 (56)	207 (9)	445 (49)
Final R_cryst_	0.152 (0.114)	0.314 (0.323)	0.249 (0.244)
Final R_free_	0.216 (0.183)	0.426 (0.406)	0.299 (0.355)
R.M.S. deviations			
Bonds (Å)	0.0007	0.005	0.004
Angles (°)	1.453	1.447	1.254
Average *B* factors (Å^2^)	19.58	32.8	20.4
Protein	20.61	11.44	21.44
Ligands	37.73	27.46	37.24
Ions	22.99	33.37	40.32
Waters	29.96	28.35	28.01
Ramachandran plot			
Most favoured (%)	98.43	88.98	96.85
Allowed (%)	1.57	11.02	3.15

Statistics for the single hit crystaland the multi-hit (first hit and second hit) data sets are presented. Values for the outer shell are given in parentheses.

## Discussion

The use of different jet speeds during the experiment enabled an analysis of the speed of recovery of the liquid jet after the jet explosion. The reliable recovery of the liquid jet between consecutive pulses at the jet speeds used in this experiment has been previously reported in the literature^1,12^. Briefly, the gap size used in this experiment is much smaller than the beam diameter. Thus, if the crystal is hit within the tail region of the beam (which is the case for the first hits), the gap will not reach the crystal before the next pulse arrives. Hence, the results presented here confirm previous reports that under standard experimental conditions, the pulse power, beam size, jet diameter, and jet speed, can be chosen to avoid any interaction of the crystal with the expanding front of the opening gap formed by a previous pulse^1,12,47^.

The results from this experiment confirm that quality structural data can be collected from crystals that are hit more than once by a MHz XFEL pulse using a flow focusing injector. In fact, the second hit data set could be refined to a higher resolution (2.1 Å) compared to the first hit data set (3.1 Å). This allows for the optimisation of multi-hit SFX experiments since the height of the beam profile can be widely tuned to match the distance travelled by the crystals between consecutive X-ray pulses. The multi-hit structures from the first and second hits in this experiment demonstrate that high-resolution data (e.g., <2.5 Å), can be collected from micro-crystals with per pulse dose rates less than that generally required to induce RT radiation damage. For the second hit data analysed, the data was of high quality, comparable to that of the single hit crystals demonstrating that radiation damage did not affect the structure. However, the first hit data could not be solved to the equivalent resolution, having a lower percentage completeness (33%) in the 2.1 Å resolution shell which can be explained by the asymmetry of the outermost tails of the beam when the beam was at its largest size. Given the aim here was to generate a structure, the peak finding algorithm parameters were not optimised specifically for identifying weaker peaks. Therefore, by further optimising the hit-finding it may be possible to identify additional peaks in the higher resolution shell for the first hit data and possibly even identify third hits for the slowest jet speeds. The same experiment is not currently possible at lower repetition rate sources using flow focusing injectors, but as more high-repetition rate XFEL sources come online the opportunity to compare time-resolved data collected from the same crystal with femtosecond intra-pulse and sub-microsecond inter-pulse timing opens possibilities for studying molecular dynamics. In 2019, the focusing optics at the SPB/SFX beamline at the European XFEL were upgraded enabling the production of a long vertical line-focus aligned to the liquid jet providing better setup optimisation for this type of experiment.

The time interval between consecutive pulses at the European XFEL is very short when compared to other non-MHz XFEL sources, however sub-microsecond pulse spacing is clearly still long on the time-scale of radiation damage^48–50^. Two methods were used to independently determine the dose absorbed by the crystal during a single pulse. The RADDOSE-3D version 4 (X-FEL)^39^ gave an estimated dose of 0.2 MGy, taking into account the photo electron escape and the FW of the beam. The second method gave an estimate of 0.165 MGy maximum dose per pulse received by the crystal within the beam using the approach of Marman et al.^51^. which includes photo electron escape and in addition specifically the absorbed dose as a function of the crystal position within the incident beam (Supplementary Fig. 5). Both methods produced dose estimates similar to those published under the same experimental conditions, (0.5 MGy reported by Weideron et al.^1^). We note that the primary reason the absorbed dose per pulse is lower here is the fact that the FW beam size is larger in the present case to provide a large enough X-ray interaction region to generate multiple hits. At the XFEL it has previously been reported, due to the short pulse duration, the per-pulse radiation damage limit, even at room temperature, could increase to the range of 30–150 MGy^4,52,53^ as it can outrun the slower process contributing to radiation damage. However, the effects of radiation damage at the XFEL as a function of pulse duration is very much an active and ongoing area of research^6,52,54,55^ and depends both on the dose rates as well as the molecular details of the sample^56^. It is also known that under cryo-conditions protein crystals can typically withstand a radiation dose of 30 MGy^57,58^, at RT (which we have assumed here) the radiation dose limit decreases by approximately two orders of magnitude, as a result of the diffusion of free radicals^59–61^. Hence in the analysis presented here, we have adopted the most conservative view that the per pulse absorbed dose must be below 0.2 MGy^57^ in line with RT experiments conducted at the synchrotron before radiation damage is to occur. These values fall within the dose estimates obtained for this crystal system where the dose of the crystal in the tail regions can be as low as 0.04 MGy (Supplementary Fig. 5) and as high as 0.2 MGy central to the beam.

Our model for how a single crystal is able to produce multiple diffraction patterns has been validated by the observed changes in the measured integrated intensity profiles and explains why, for example, the second hit for the 42 m/s jet has a similar intensity profile to the single hit crystals. It also accounts for the fact that for the faster jet speeds the intensity profiles of the first and second hits are similar. Another interesting observation was that even within the outermost tails of the XFEL beam, where the absorbed dose is around 2 orders of magnitude lower than in the beam centre, there is still sufficient intensity to generate high-resolution diffraction data from crystals <10 µm in diameter. This is consistent with previous published results from the LCLS^4^.

**Fig. 6:**
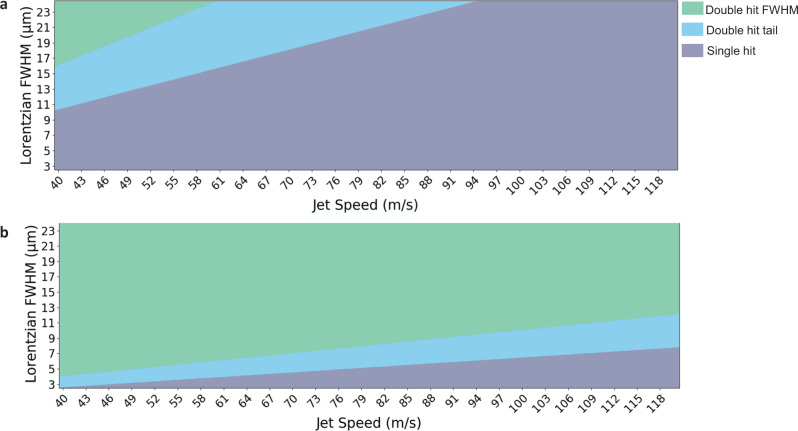
Parameters for optimizing the collection of double-hit data at the European XFEL. **a** European XFEL repetition rate of 1.1 MHz during this experiment and (**b**) European XFEL repetition rate of 4.5 MHz. The green shaded area indicates parameter combinations that will result in double-hits that allow the second hit to occur within the horizontal FWHM of the beam; the blue shaded area indicates parameter combinations that will result in double-hits that allow a second hit to occur within in the tail region of the beam, and the grey shaded area indicates parameter combinations that will result in only single hits. This analysis is independent of crystal size (i.e., crystal centre-to-crystal centre hits).

The experimental setup for multi-hit SFX can be easily and quickly realised on the SFX/SBP beamline at the European XFEL, multi-hit SFX is an option available to all users. Based on our model for how multiple hits occur we can develop a set of parameters for users that can be employed to either maximise or minimise multiple crystal hits. The critical parameters for multi-hit SFX are the beam size, jet speed, and pulse structure. For these measurements the AGIPD detector was operating at a full frame read-out of 1.1 MHz using 202 calibrated memory cells. This potential bottleneck in data collection has since been addressed and the detector is currently capable of operating at a maximum full-frame readout of 4.5 MHz (the European XFEL main frequency) using 351 calibrated memory cells. The inter-pulse spacing used for the current experiment was 886 ns (1.125 MHz), however, we can use our model to predict how multi-hit SFX would work at 222 ns (4.5 MHz). A comparison of the two regimes for the European XFEL operating at 1.1 MHz and 4.5 MHz is provided in Fig. 6.

In addition to the integrated intensity, we also studied the resolution of the diffraction data from both single and double hits. A comparison of the second hit and single hit patterns shows a difference in the resolution of 1 Å which can be explained by the beam profile and SNR detection levels. This is also supported by the structural analysis where both the first and second hit structures do not exhibit any noticeable signs of radiation damage. Hence, the key finding here is that the diffraction data, statistics, structural analysis, and radiation damage calculations all point to the same conclusion: that the crystal does not appear to experience measurable radiation damage during the first hit by the XFEL beam for this crystal system.

The availability of MHz XFEL sources has created many opportunities for exploiting their unique pulse structure^12,62^. The approach described and demonstrated here, measuring structure from the same single crystal in-flight with consecutive MHz XFEL pulses, opens up the possibility of correlating and analysing structural dynamics on timescales ranging from 222 ns (4.5 MHz) to 1.8 µs (approximated based on a crystal travelling at 42 m/s through an interaction region with a mean beam width of 65.8 µm). This covers a range of molecular dynamics of interest to structural biology including helix motions, side chain rotations, and protein folding and unfolding. The experimental conditions used for the current experiment are readily achievable at the European XFEL and have been used for a recent publication involving tr-SFX^63^ demonstrating that multi-hit SFX is a viable option for current and future users of this facility. Two types of experiments have been established as benefitting from this mode of operation, the first is chemically triggered SFX where a mix-and-inject setup is used on the beamline. Using multi-hit SFX would allow the dynamics of each single crystal to be probed at a minimum of two timepoints with sub-microseconds separation. The second type of experiment to benefit from multi-hit SFX is optical pump/X-ray probe experiments where two structures (one ‘dark’ with the laser off, and one ‘light’ activated with the laser on) can be obtained from the same crystal. Whilst for proteins that are particularly radiation sensitive, multi-hit SFX may not be an option, the fact that the two independent structures from the first and second hit measured here do not exhibit any detectable signs of radiation damage is encouraging. Based on the criteria we have established here to observe multiple hits using MHz XFEL sources the current work paves the way to multi-hit SFX becoming an established mode of operation for users.

## Methods

### Sample preparation

Microcrystals of hen egg white lysozyme (HEWL) were grown by rapid-mixing batch method as describe in Wiedorn et al. (2018)^1^. The lysozyme (126 mg/mL in 50 mM acetate buffer pH 3.5) crystal were grown in 1 M sodium chloride, 40%(v/v) ethylene glycol, 15%(w/v) poly ethylene glycol 4000, 50 mM acetate buffer pH 3.5 and varied in size between 6 × 6 × 6 µm^3^ and 8 × 8 × 8 µm^3^ as characterised via an optical microscope.

### SPB/SFX instrument and sample delivery

Experiments were performed at the SPB/SFX instrument at the European XFEL in September 2017 as part of European XFEL experiment p2012 as describe in Wiedorn et al. (2018)^1^. The SPB/SFX beam was focused using Compound Refractive Lenses (CRLs). Lysozyme (HEWL) crystals were delivered to a 1.125 MHz XFEL beam using a 3D printed GDVN^33,34,64^ nozzle with 60 µm gas and 50 µm liquid orifices. Design and fabrication details can be found in Knoska, J. et al.^65^ Various sample delivery jet speeds ranging between 40-102 m/s, were tested during the experiment. Data was measured using an AGIPD 1 M located 0.12 m downstream of the sample interaction region. All the data generate from these speeds were merged to generate the first and second hit data sets. The majority of the data was generated from three speeds, 42 m/s, 78 m/s and 102 m/s, and these were used to generate statistics for the beam profile. The two slowest jet speeds were checked experimentally in lab by dual-pulse imaging^1,65^, whilst the fastest jet speed (102 m/s) was theoretically determined (see Supplementary Information). A summary of the liquid and gas flow rates and their equivalent jet speeds are shown in Table 1, alongside the theoretically calculated 102 m/s jet speed.

### X-ray data analysis and processing

Experiment progress was monitored online using OnDA^66^ or serial crystallography reading data in real time from the European XFEL control system Karabo^67^ using the Karabo bridge^68^, as outline in Wiedorn et al. (2018)^1^. SFX data processing was done as previously outlined in Wiedorn et al. (2018)^1^ using CrystFEL v.0.6.3 on peaks found by Cheetah using the indexing packages MOSFLM^69^, DirAx^70^ and asdf^71^. The SNR value was optimised for each dataset to allow optimal peak detection^72^.

### Identification of multi-hits in the data

The CrystFEL stream files with the indexed crystal data^1^ were used to identify the crystals classified as multi-hits for the different injector speeds. The stream files were analysed for multi-hits using the Whirligig script from the CrystFEL crystallography suite^45^, which defines crystals hit twice as those that had similar crystallographic orientations (<5° change in angle between each pair of basis vectors) in consecutive frames. Based on this analysis we confirmed that a proportion of single crystals were hit twice, classifying them as multi-hit crystals. In addition, the lengths of the basis vectors had to be within 10% agreement to qualify as a multi-hit. Filtering using the Whirligig program based on these criteria alone does not account for the pulse train format of the European XFEL. Therefore, python code was developed to filter out any adjacent frames that were not from the same pulse train. This data was further sorted into three separate categories: data from crystals hit just once, data from the first hit of crystals hit twice (designated first hit), and data from the second hit of crystals hit twice (designated second hit). These three stream files were then used as the input files for further analysis in Python, using multiple parameters (integrated intensity, a*, b*, c*, *h*, *k*, *l*) within the stream files to calculate 1/d verses normalised integrated intensity plots.

Python code was also used to analyse the CrystFEL stream files, where the change in orientation between all consecutive images was calculated in an identical manner to the Whirligig program. The output was plotted as a histogram, showing the change in angle (degrees) for consecutive images, to confirm those consecutive hits that were selected as the double hit data set were truly from the same crystal.

### Analysis of data quality

Merging and scaling of the Bragg peaks were performed using Partialator in the CrystFEL suite version 8^45,71^. Figures of merit were calculated using compare_hkl (Rsplit, CC_1/2_, CC*) and check_hkl (SNR, multiplicity, completeness), that are also part of the CrystFEL suite^45,71^. To generate a complete dataset to compare the first hit with the second hit on the same crystal, data collected from all jet speeds (40-102 m/s) were merged to form the multi-hit structural data sets. Data from a single speed (42 m/s) was used to analyse and solve the single hit structure. The statistics for the single hits, first hits and second hit data sets are presented in Tables 2, 3 and the Wilson plots for all the data sets are shown in the Supplementary Information (Supplementary Fig. 4).

### SFX structure determination

Structure refinement was performed in Collaborative Computational Project 4 interactive version 2 (CCP4i2)^73^ using the MTZ output from CrystFEL. A solvent free version of lysozyme (PDB accession code 6FTR) was used as the initial starting model for molecular replacement in Phaser^74^ and the R_free_ flags were generated (utilizing 10% of the data) followed by iterative cycles of Refmac5^75^ refinement and rebuilding of the model in Coot^76^. The MolProbity^77^ and Xtriage (Phenix)^78^ tools were used to validate the model. Three structures were solved, single hit structure (PDB accession code 6WEB), first hit structure (PDB accession code 7TUM) and second hit structure (PDB accession code 6WEC) which have been deposited into the PDB. To compare the first (7TUM) and second hit (6WEC) structures, difference electron density maps (DED)^9,16,79,80^, were generated in CCP4i2^73^. The two data sets were scaled, and the difference amplitudes determined by subtracting the observed structure factor amplitudes of the first hit data set from those of the second hit data set. The DED maps were then calculated by using the difference amplitudes and phases from the first hit data. The DED maps generated positive electron density (green, indicating the presence of increased density in the second hit data compared to the first hit data) and negative electron density (red, indicating decreased density in the second hit data compared to the first hit data) areas scaled to ±3σ contour levels. Figure 5a, c highlights the quality of the electron density maps surrounding the active site and a di-sulphide bond in lysozyme (which typically are more sensitive to the effects of radiation damage).

### X-ray beam profile analysis

The nominal energy for the SFX data collection was 9.232 keV. The beam size and beam profile were estimated based on 6773 individual YAG images collected using the in-line microscope positioned within in the chamber. The optical images were generated from single shots using a 15 µm thick Ce:YAG screen (see Supplementary Fig. 3 and “Methods”). The Point Spread Function (PSF) of the YAG was determined, based on published estimates^81^ (see Supplementary Fig. 3), to be 2 µm. Using an Edmund optics standard, the optical microscope resolution was determined to be 8 µm. A Lorentzian distribution was fitted to the optical images using Python code. The actual beam size was determined via a convolution, taking into account both the PSF for the YAG and optical microscope. A FWHM (FWHM = 2ɣ) for each image was determined from a Lorentzian fit to the beam profile using a 3 × 3 median filter and a 7.5% noise threshold applied to the data. A FW value of 82.38% (FW = 7.04ɣ) was also determined for each image, and the overall mean, minimum and maximum FW calculated for the complete data set. The mean, minimum, and maximum FWHMs and FW were calculated. Note that the experimentally determined beam size based on analysis of the occurrence of multi-hits as a function of jet speed (Fig. 1) was consistent with the X-ray beam size determined optically.

### Crystal transit through the X-ray beam

The distance travelled by the crystal through the X-ray beam can be calculated based on the jet speed (see Table 1) and beam size. Using the upper limit on the measured size of the lysozyme crystals (i.e., 8 × 8 × 8 µm^3^) and the lowest possible jet speed (accounting for a 5% uncertainty—see Table 1) gives the minimum distance a single crystal could travel whilst still interacting with two consecutive X-ray pulses, spaced 886 ns apart. Assuming that at least 1 µm of the crystal needs to interact with the X-ray beam to generate a diffraction pattern, the minimum distance travelled by a single crystal hit twice by the XFEL beam was calculated. For the fastest jets, multi-hits were only possible in the presence of the maximum FW of the beam.

### Radiation dose calculations

To determine the dose the crystal receives during its interaction with the X-ray pulse two independent methods were used. The first method is based on Monte-Carlo modelling of the primary photoelectron trajectories, taking in account any photoelectron escape from the crystal that might occur subsequent to the crystal interacting with the X-ray pulse. This approach to determining the dose absorbed by the crystal was adapted from a discrete simulation of radiation damage model based on Marman et al.^51^. The X-ray beam was modelled as a symmetric 2-dimensional Lorentzian distribution with a full width of 65.5 µm (accounting for 99% of the X-ray flux). The total X-ray interaction area accounted for in the model was 100 µm in diameter allowing the crystal to be simulated prior to entering and thus only partially exposed to the X-ray beam. The spatial resolution of the model was 0.1 µm. This was sufficient to allow us to determine the dose received by the crystal at different points within the beam (i.e., within the tail region or within the central portion of the beam) as it travels through the X-ray interaction region. The Lorentzian flux distribution and the appropriate X-ray cross-sections for photoionisation, elastic scattering, and Compton scattering at this X-ray energy were used to calculate the relevant interaction rates at any given point within the beam^82^. The crystal was modelled as a square (8 µm × 8 µm); and was assumed to pass through the centre of the beam travelling perpendicular to the y-axis. The absorbed dose was calculated at 33 independent positions along the x-axis. These positions corresponded to the crystal moving from the edge of the beam to its centre in 1 μm steps; therefore 7 of the 33 positions corresponded to a partial hit, where part of the crystal remained unexposed and the remaining 26 positions corresponded to full exposure at varying incident flux densities. At each position, the dose absorbed by the exposed portion of the crystal was calculated. A summary of this approach to calculating the radiation damage is presented in Supplementary Fig. 5. We note that in the tail regions of the beam the dose received by the crystal can reduce by as much as two orders of magnitude. As an independent confirmation of the first approach, a second method, based on RADDOSE-3D version 4^39,83^ using the XFEL option, assuming a Gaussian distribution for the beam (no collimation) and maximum full width of beam was used and the crystal was placed central to the beam. In summary, using these two independent methods, the dose absorbed by the crystal during both the first and second hits was calculated.

## Supplementary information

Supplementary Information

## Data Availability

Source data have been deposited with the Coherent X-ray Imaging Databank (CXIDB) with reference number CXIDB-ID80 (www.cxidb.org/id-80.html) for run numbers r0066-r0087, r0145-r0150 and r0153. The structures have been deposited in the Protein Data Bank (PDB), accession codes 6WEB (single hit structure), 7TUM (first hit structure) and 6WEC (second hit structure). Other data are available from the corresponding authors upon reasonable request.
