# Supplementary material for: Megahertz pulse trains enable multi-hit serial femtosecond crystallography experiments at X-ray free electron lasers
Source: Nat Commun. 2022 Aug 11;13:4708. doi: 10.1038/s41467-022-32434-6 (PMC9372077; doi:10.1038/s41467-022-32434-6)
Supplement: Supplementary file 1 — Supplementary Information [file 41467_2022_32434_MOESM1_ESM.pdf]

Megahertz pulse trains enable multi-hit serial femtosecond crystallography experiments at X-ray Free Electron Lasers

Supplementary Information

Supplementary Methods

Injector speed calculations

The speed of a free jet can be expressed as

$$v = \sqrt{2\Delta P/\rho}\ . \qquad (1)$$

Where v is the jet speed (m/s), $\Delta P = p_i - p_f$ is the gas pressure drop over the discharge orifice with p_i the pressure (N/m^2) before the jet and p_f the ambient pressure after the jet, and ρ is the density of the fluid (kg/m^3). The value of the fastest jet speed was calculated assuming that the fluid density is constant for all jets. Then, using the fact that the gas flow rate, g, is proportional to ΔP, the ratio of any two different jet speeds (e.g. v_1 and v_2) is given by the square root of the ratio of the corresponding gas flow rates, i.e.

$$\frac{v_1}{v_2} = \sqrt{{}^{g_1}/_{g_2}} \qquad (2)$$

Using equation 2 it is thus possible to solve for the (unknown) value of the fastest jet speed using the experimentally measured jet speed and gas pressure for a slower jet and the measured gas flow rate for the fastest jet speed. E.g. using the experimental values for the 78 m/s jet yields a theoretical value of $v_2 = 78/\sqrt{50/85} = 102\ m/s$ for the fastest jet speed (see Table 1 in main text).

Supplementary Figures

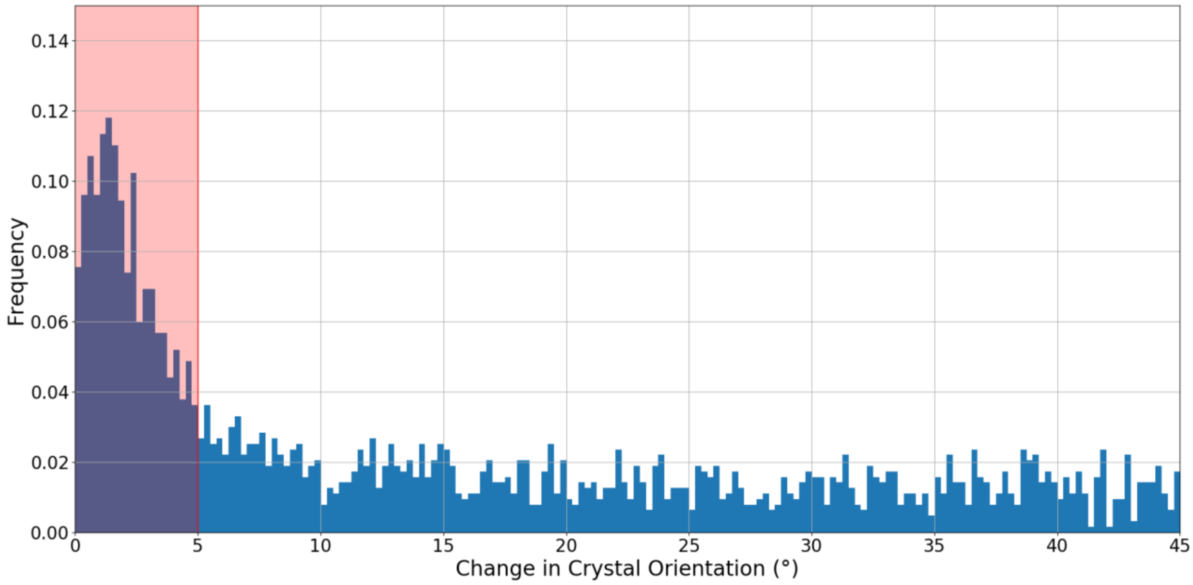

Supplementary Figure 1. Image frequency as a function of the relative change in crystal rotation for b axis. It is characterized by reciprocal space vector $\vec{b}$, between consecutive diffraction measurements (separated by 886 ns) within the pulse train. The jet speed = 42 m/s. The increase in frequency with a change in orientation of less than 5 degrees is due to the occurrence of double hits.

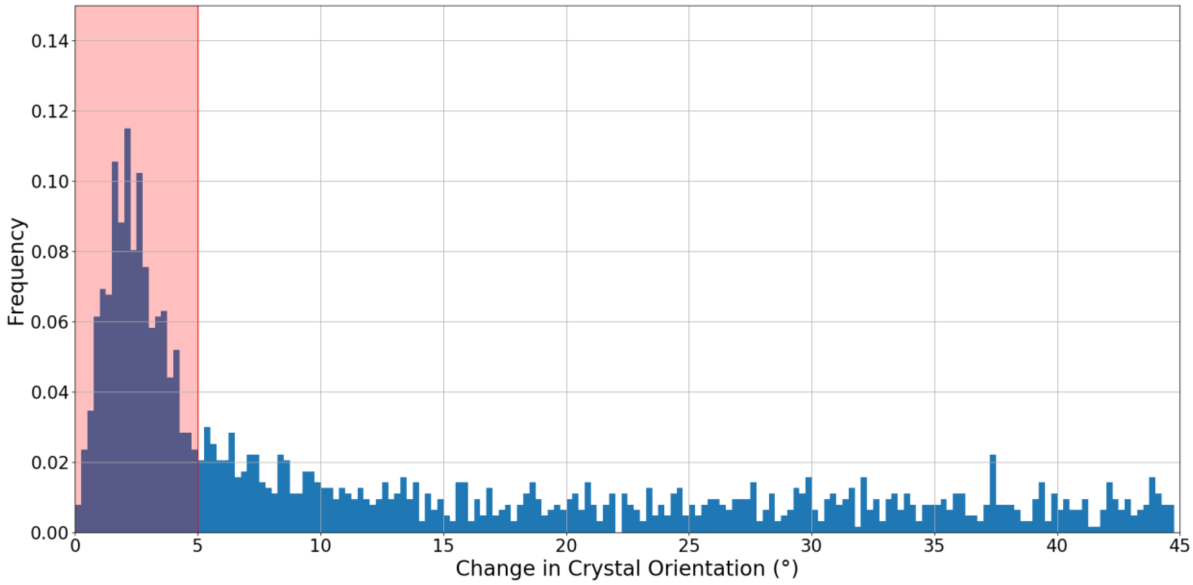

Supplementary Figure 2. Frequency of images as a function of the relative change in crystal rotation for c axis. It is characterized by reciprocal space vector $\vec{c}$, between consecutive diffraction measurements (separated by 886 ns) within the X-ray pulse train. The

jet speed was 42 m/s. An increase in the frequency of images with a change in orientation of less than 5 degrees can be seen due to the occurrence of double hits.

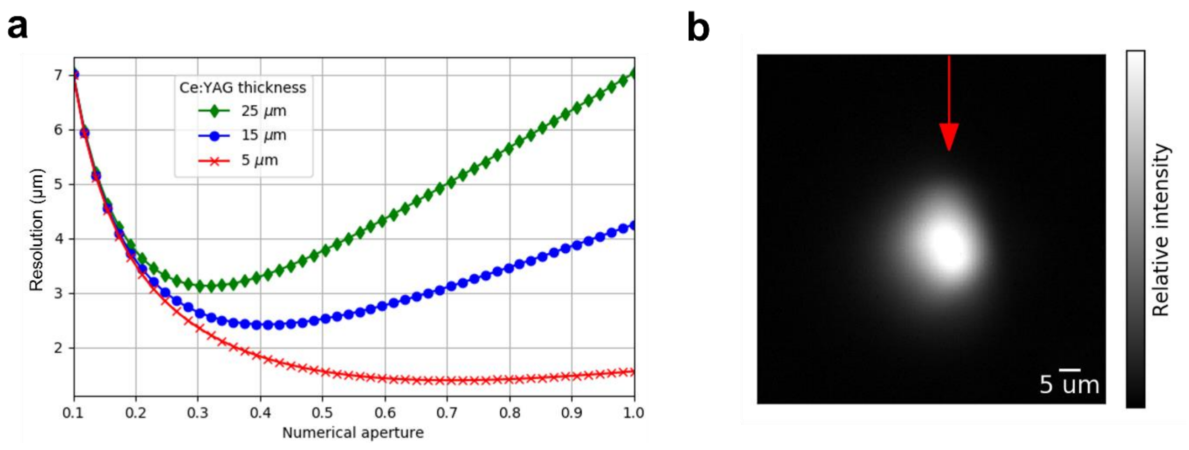

Supplementary Figure 3. YAG measurements. a Resolution of a CE:YAG as a function of Numerical Aperture (NA) for 3 different thicknesses. The plot is based on data from Koch et. al. [1]. The NA for the optical imaging system for the SPB beamline at the EuXFEL is between 0.3 to 0.4, providing a resolution estimate between 2-3 µm for the 15 µm YAG thickness. **b** Average YAG image determined during the experiment with the red arrow indicating the jet directions.

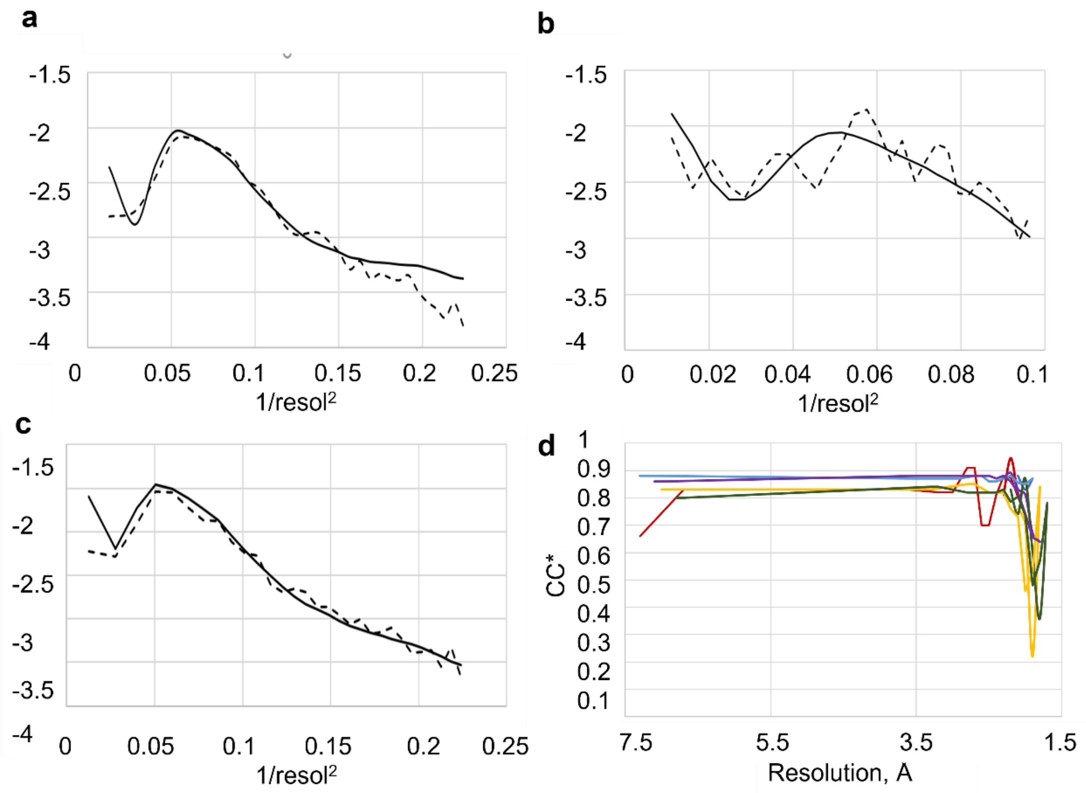

Supplementary Figure 4. Statistical plots for the lysozyme structure. Wilson plot of merged data using Phenix for: **a** single hits, **b** first hits, and **c** second hits. The dotted line indicates (In(I/I_th) and the solid line represents (Reference protein). **d** CC* for data separated into single hits (blue), first hits (red) and second hits (yellow) as well a correlation of merged data of the second hit relative to the first hit (green) and single hit (purple) data sets, indicating they are very similar. The first hit structure is red and the second hit structure is green.

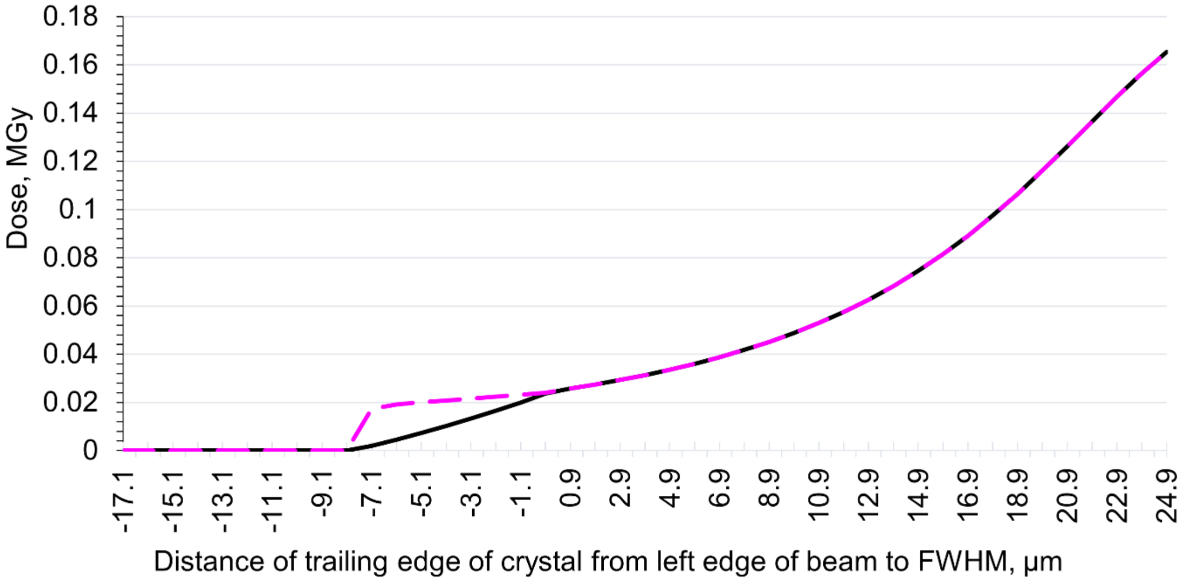

Supplementary Figure 5. Plot showing the dose a crystal would receive as it enters the first half of the X-ray beam, left of the full width half maxima (FWHM). The dose is calculated based on 8 × 8 × 8 μm^3 crystal size using the approach of Marman et. al.[2] Both the whole crystal dose (dotted pink line) and the dose only for the exposed part of the crystal are shown (solid black line).

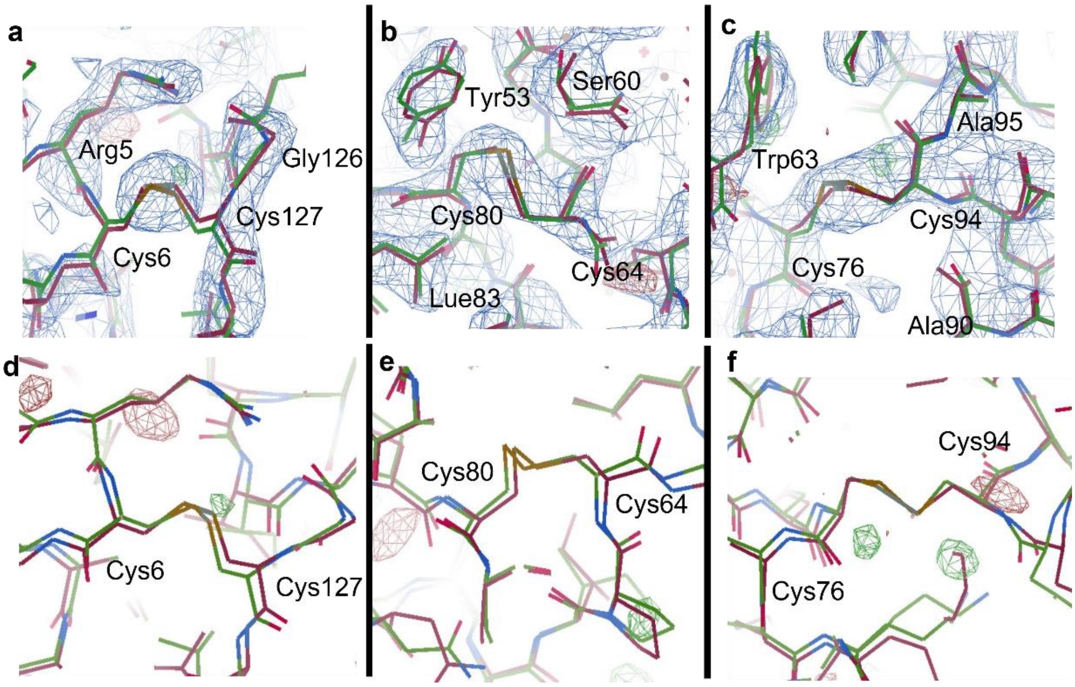

Supplementary Figure 6. Density maps around three di-sulfide bonds in the lysozyme structure. a Cys6 and Cys127, **b** Cys80 and Cys64 and **c** Cys76 and Cys94 and their corresponding Difference Electron Density (DED) maps, **d, e,** and **f**. The 2Fo-Fc map at 1σ is shown (blue) and Fourier difference (Fo-Fc) maps at 3σ. Green indicates positive density and red negative density.

Supplementary References

1 Koch, A., Raven, C., Spanne, P. & Snigirev, A. X-ray imaging with submicrometer resolution employing transparent luminescent screens. J. Opt. Soc. Am. **15**, 1940-1951 (1998).

2 Marman, H., Darmanin, C. & Abbey, B. The Influence of Photoelectron Escape in Radiation Damage Simulations of Protein Micro-Crystallography. Crystals **8**, 267 (2018).
